# The global progress of soil-transmitted helminthiases control in 2020 and World Health Organization targets for 2030

**DOI:** 10.1371/journal.pntd.0008505

**Published:** 2020-08-10

**Authors:** Antonio Montresor, Denise Mupfasoni, Alexei Mikhailov, Pauline Mwinzi, Ana Lucianez, Mohamed Jamsheed, Elkan Gasimov, Supriya Warusavithana, Aya Yajima, Zeno Bisoffi, Dora Buonfrate, Peter Steinmann, Jürg Utzinger, Bruno Levecke, Johnny Vlaminck, Piet Cools, Jozef Vercruysse, Giuseppe Cringoli, Laura Rinaldi, Brittany Blouin, Theresa W. Gyorkos

**Affiliations:** 1 Department of Control of Neglected Tropical Diseases, World Health Organization, Geneva, Switzerland; 2 Expanded Special Programme for Elimination of Neglected Tropical Diseases, World Health Organization, Regional Office for Africa, Brazzaville, Congo; 3 Neglected, Tropical and Vector Borne Diseases, World Health Organization Regional Office for the Americas, Washington, United States of America; 4 Neglected Tropical Disease Control, World Health Organization, Regional Office for South-East Asia, New Delhi, India; 5 Malaria, NTDs and other Vector-Borne Diseases, World Health Organization, Regional Office for Europe, Copenhagen, Denmark; 6 Control of Communicable Diseases, World Health Organization, Regional Office for Eastern Mediterranean, Cairo, Egypt; 7 Malaria, other Vectorborne and Parasitic Diseases, World Health Organization, Regional Office for Western Pacific, Manila, The Philippines; 8 Department of Infectious–Tropical Diseases and Microbiology, IRCCS Sacro Cuore Don Calabria Hospital, Negrar, Verona, Italy (WHO Collaborating Centre ITA-102); 9 Swiss Tropical and Public Health Institute, Basel, Switzerland (WHO Collaborating Centre SWI-71); 10 University of Basel, Basel, Switzerland; 11 Department of Virology, Parasitology and Immunology, Faculty of Veterinary Sciences, Ghent University, Merelbeke, Belgium (WHO Collaborating Centre BEL-42); 12 Laboratory of Parasitology and Parasitic Diseases, Department of Veterinary Medicine and Animal Production. University of Naples, Naples, Italy (WHO Collaborating Centre ITA-116); 13 Department of Epidemiology, Biostatistics and Occupational Health, McGill University, Montreal, Canada (WHO Collaborating Centre CAN-88); NIH-NIRT-ICER, INDIA

## Abstract

Soil-transmitted helminth (STH) infections are the most widespread of the neglected tropical diseases, primarily affecting marginalized populations in low- and middle-income countries. More than one billion people are currently infected with STHs. For the control of these infections, the World Health Organization (WHO) recommends an integrated approach, which includes access to appropriate sanitation, hygiene education, and preventive chemotherapy (i.e., large-scale, periodic distribution of anthelmintic drugs). Since 2010, WHO has coordinated two large donations of benzimidazoles to endemic countries. Thus far, more than 3.3 billion benzimidazole tablets have been distributed in schools for the control of STH infections, resulting in an important reduction in STH-attributable morbidity in children, while additional tablets have been distributed for the control of lymphatic filariasis. This paper (i) summarizes the progress of global STH control between 2008 to 2018 (based on over 690 reports submitted by endemic countries to WHO); (ii) provides regional and country details on preventive chemotherapy coverage; and (iii) indicates the targets identified by WHO for the next decade and the tools that should be developed to attain these targets. The main message is that STH-attributable morbidity can be averted with evidence-informed program planning, implementation, and monitoring. Caution will still need to be exercised in stopping control programs to avoid any rebound of prevalence and loss of accrued morbidity gains. Over the next decade, with increased country leadership and multi-sector engagement, the goal of eliminating STH infections as a public health problem can be achieved.

## Author summary

Intestinal worm infections are extremely frequent in areas where sanitation, access to clean water, and hygiene measures are poor. These infections negatively affect the nutritional status and development of children and women. The World Health Organization (WHO) recommends the periodic administration of anthelmintic drugs as one of the components of a control strategy to eliminate these infections. Since 2010, WHO has managed two donations of anthelmintics (i.e., albendazole and mebendazole) that are provided to ministries of health in endemic countries. The present paper summarizes anthelmintic treatment coverage data over a 10-year period (2008–2018) from over 690 reports (reporting for more than 3.3 billion tablets) submitted to WHO by ministries of health. It also reports on the results of surveys, which have been conducted in several countries to evaluate the impact of the drug distribution. Lastly, it presents global objectives for the control of intestinal worm infections, to be achieved by 2030, as identified by a panel of WHO experts and collaborators. In conclusion, the program for the control of intestinal worm infections has steadily progressed between 2008 and 2018 and achieving a coverage level of over 60% in school-age children. The health consequences of these infections have also been significantly reduced. With sustained program implementation, the elimination of these worm infections as a public health problem might be achieved by 2030.

## Introduction

### Soil-transmitted helminthiasis and the control strategy recommended by the World Health Organization

Soil-transmitted helminth (STH) infections are the most widespread of the neglected tropical diseases, primarily affecting marginalized populations in low- and middle-income countries. Indeed, more than one billion people are currently infected with one, or several, of the most common STH species. These include *Ascaris lumbricoides*, *Trichuris trichiura*, and the two hookworm species, *Necator americanus* and *Ancylostoma duodenale* [1,2]. Each of these four species has distinct characteristics, but they are considered as a single group because of similarities in transmission dynamics and the prevention and control measures needed.

To achieve sustained control of STH prevalence, infection intensity, and morbidity, the World Health Organization (WHO) recommends an integrated approach, which includes access to appropriate sanitation, hygiene education, and preventive chemotherapy (PC) [3]. PC consists of the periodic administration of anthelmintic drugs (in the case of STH, either albendazole or mebendazole) to high-risk populations in endemic areas. Parasitological surveys are conducted to identify the high-risk populations and geographic areas where PC is needed. If the baseline STH prevalence in school-age children equals or exceeds 20%, PC is recommended to be implemented–without the need to assess individual infection status [4,5]. Once the baseline prevalence has informed the frequency of PC (i.e., either once or twice a year), progress is ideally monitored approximately every 2–3 years. After 5 years of annual PC implementation, the PC program is re-evaluated to determine next steps [4]. This strategy is cost-effective and periodic surveys provide sufficient information for effective program implementation and timely adaptation. An initial target coverage of 75% in the high-risk group of school-age children was set by WHO in 2001 [6]. This coverage target is now also applied to the two other groups at highest risk of STH-related morbidity: preschool-age children and women of reproductive age [4,7].

### Countries and populations at risk of STH

In 2010, the total number of countries and territories endemic for STH was estimated to be 112 [8]. With on-going collection of epidemiologic data and refinement in estimation parameters, this number was updated to 96 STH-endemic countries in need of PC in 2018 [9]. Three population groups are identified by WHO as being at highest risk for STH-attributable morbidity, namely (i) preschool-age children; (ii) school-age children; and (iii) women of reproductive age.

For each STH-endemic country, WHO confirms the number of children in need of PC at the national level and for each district, adjusting these numbers every year according to any new epidemiologic information provided. The sum of country numbers informs estimates at the global level. In 2018, it was estimated that 310 million preschool-age children [9], 762 million school-age children [9], and 688 million women of reproductive age (including 69 million pregnant women) [10] were at risk of STH infection, and hence, in need of PC.

### Anthelmintic drug donation for STH control

Since 2010, WHO has coordinated two large donations of benzimidazoles for the control of STH infection: albendazole and mebendazole. GlaxoSmithKline (manufacturing albendazole) and Johnson & Johnson (manufacturing mebendazole), together, provide a total of 600 million tablets per year. Between 2010 and 2020 approximately 3.3 billion tablets of benzimidazole (1.9 billion albendazole and 1.4 billion mebendazole) have been donated for the control of STH in school-age children.

Initially, these donations were strategically targeted for school-age children only and they provided a consistent source of anthelmintics over the period 2010–2018. Of note, in 2019, Johnson & Johnson made its new chewable formulation of mebendazole available [11], which allowed its donation to extend to preschool-age children [12]. Every year, after submitting a report on coverage results from the previous year to WHO, countries in need of benzimidazoles for STH control may request the number of anthelmintic tablets they plan to distribute over the upcoming year. In this way, global progress has been documented and anthelmintic drug supplies managed.

### Aim and structure of the paper

In this paper, we summarize the global progress made toward PC coverage targets set by WHO (part 1); we report on the impact of PC and other STH control activities to date (part 2); and, we introduce the WHO targets and activities on the road to elimination of morbidity caused by STH infections and strongyloidiasis by 2030 (part 3).

## Part 1: Global progress in PC coverage for the control of STH infection between 2010 and 2018

### Materials and methods

Since 2010, WHO has organized a system in which any country requesting a donation of anthelmintics reports the previous year’s coverage, for preschool- and school-age children separately. Countries who do not need to request a donation are also invited to report their coverage levels to WHO. The Joint Reporting Form (JRF) is a standard form developed by WHO [13] to facilitate the reporting of coverage achieved every year by NTD control programs. It consists of an Excel file with tags for each group of parasites targeted (e.g., STH, schistosomiasis, and lymphatic filariasis), for each at-risk group, by endemic district. The form automatically calculates the coverage by district and nationally.

Over the period 2008 and 2018, three versions of the form have been developed, each subsequent version improving on its user friendliness interface but keeping the minimal essential information collected the same. Once received, these forms were cross-checked with other information available in WHO (e.g., estimated population at risk in each country, number of donated drugs, and reports from non-governmental organizations (NGOs) and other donors collaborating at local level). Whenever discrepancies occurred, the form was discussed with the national manager of the control program and, if need be, corrected. In some countries the Ministry of Health (MOH) decided to also treat children in areas that were not considered in need of PC for STH. This decision was frequently based on logistic considerations (e.g., if a distribution of praziquantel for the control of schistosomiasis was organized in a district where the STH prevalence was under 20%, and therefore not in need of PC for STH, because the marginal additional cost was considered to be minimal, it was decided to also distribute benzimidazoles at the same time). In these cases, WHO distinguishes between the “number of children reported to be treated” and the “number of children treated in areas requiring PC”. The “number of children treated in areas requiring PC” was divided by the “number of children in need of PC” in each district and country, thus enabling the calculation of relevant coverage rates at the district and country level.

### Results

WHO received 693 JRFs documenting treatment of preschool- and/or school-age children, between 2008 and 2018. The number of countries reporting treatment for preschool-age children was, on average, 63, with a range between 54 and 77 countries reporting in any one year.

#### Preschool-age children

**Fig 1:**
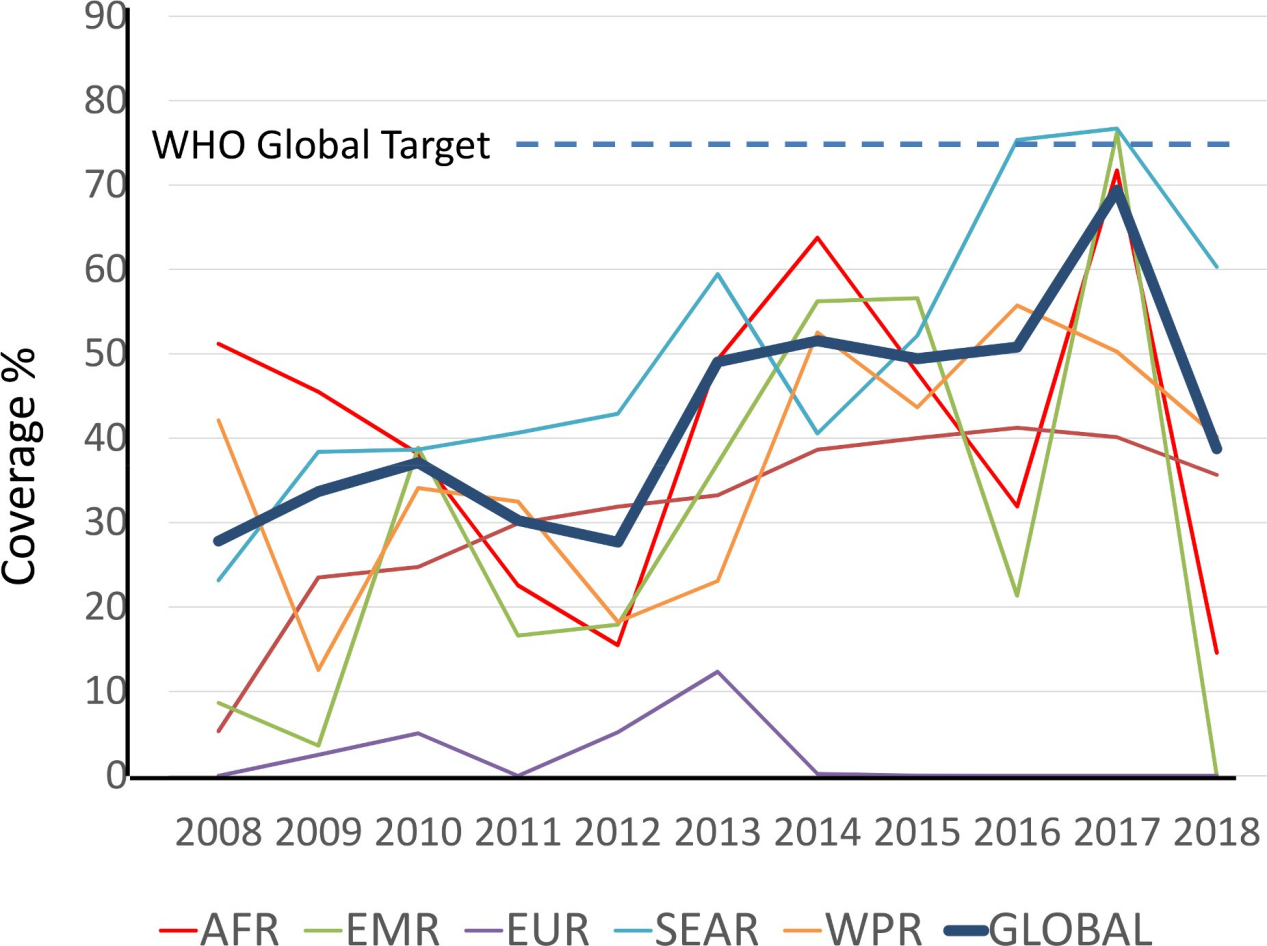
Trends in PC coverage in preschool-age children for STH between 2010 and 2018, by WHO region. Data are presented globally and stratified by WHO region (AFR = African Region; AMR = Region of the Americas; EMR = Eastern Mediterranean Region; EUR European Region; SEAR = South East Asian Region; WPR Western Pacific Region).

Despite the historic absence of a donation targeted to this age group (donations have been formally only for school-age children), there has been increasing PC coverage of preschool-age children since 2010 when WHO began to track country-led PC programs targeting this high-risk age group. Fig 1 provides details on the scaling up of the PC coverage in preschool-age children globally, and by WHO region. In addition to country reports, WHO also collects and verifies reports from NGOs that frequently distribute anthelmintics on a small scale. These NGOs are also invited to report their activities to neglected tropical diseases focal points in MOHs in order to enable a comprehensive view of the deworming interventions being undertaken in-country.

In total, 16 countries reached a coverage of ≥75% among preschool-age children in 2018. Most treatments in this age group (87%) were distributed during national “child health days” frequently alongside vaccination, vitamin A supplementation, or other public health campaigns. The remaining 13% were covered through programs for the elimination of lymphatic filariasis.

In 2018, a decline in treatment coverage was observed among preschool-age children, especially in countries of the African Region, but also in EMRO (i.e., Afghanistan) where no treatment campaign was conducted in 2018. This was probably due to difficulties encountered in the procurement of pre-qualified anthelmintics and the absence of a donation program specifically for this age group. The fact that the drugs distributed in this age group were not part of the WHO donation program further reduced the ability of WHO to collect accurate coverage data.

These difficulties are expected to be overcome with the registration, pre-qualification by WHO, and subsequent availability of chewable mebendazole and the expansion of the donation of this new formulation to preschool-age children. Consequently, coverage rates in this age group are expected to rise again.

#### School-age children

**Fig 2:**
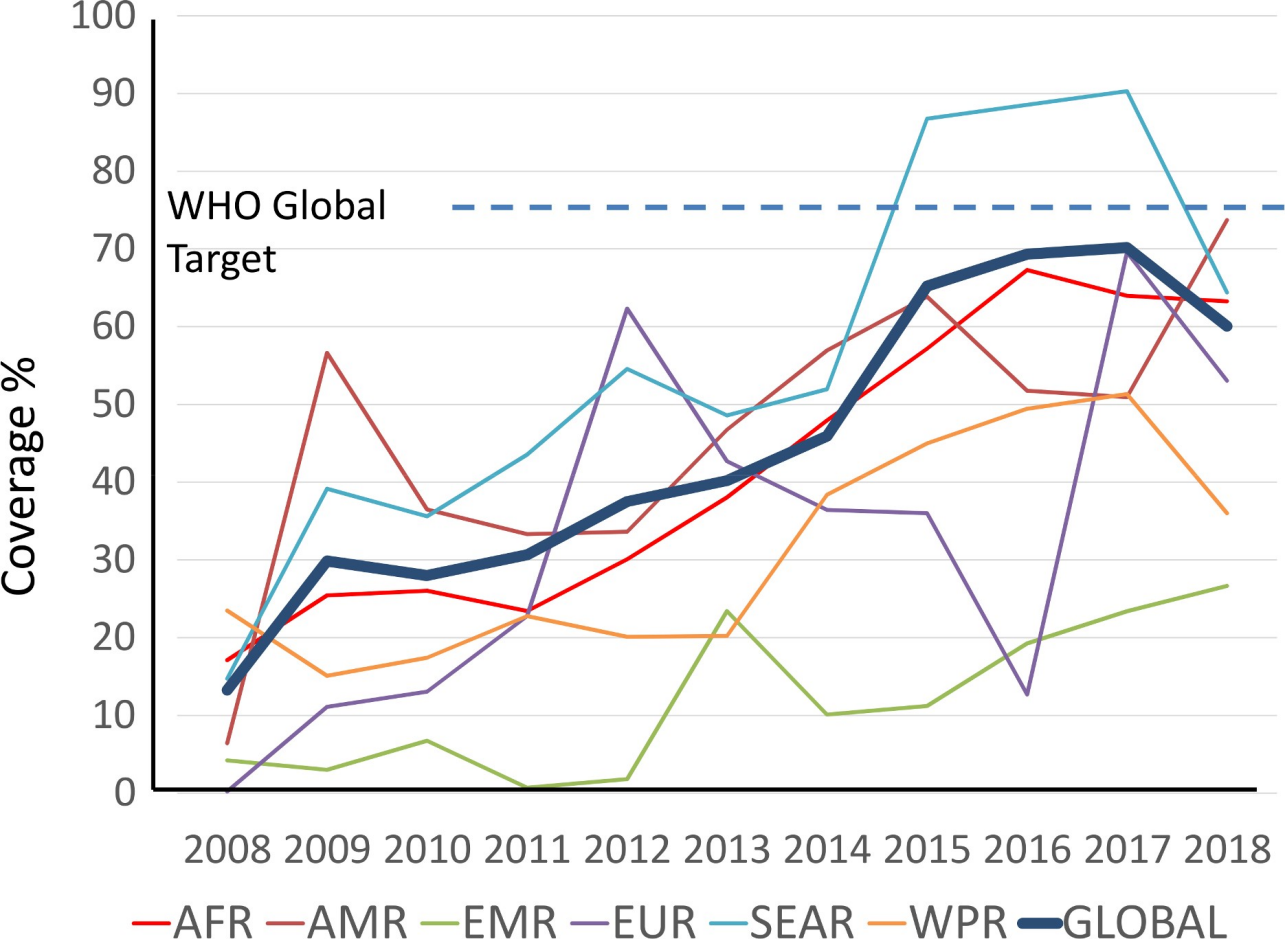
Number of school-age children in need of PC treated between 2008 and 2018, and coverage of PC in this age group.

The number of school-age children treated with PC has progressed steadily from less than 120 million in 2008 to over 450 million in 2018 (Fig 2). Most of school-age children received anthelmintics in school health programs (82%), while 18% of the children received albendazole in the context of programs for the elimination of lymphatic filariasis, the treatment administered in this program includes ivermectin in addition to albendazole, this results in increased efficacy on *T*. *trichiura* and also on treatment of individuals infected by *Strongyloides stercoralis*.

**Fig 3:**
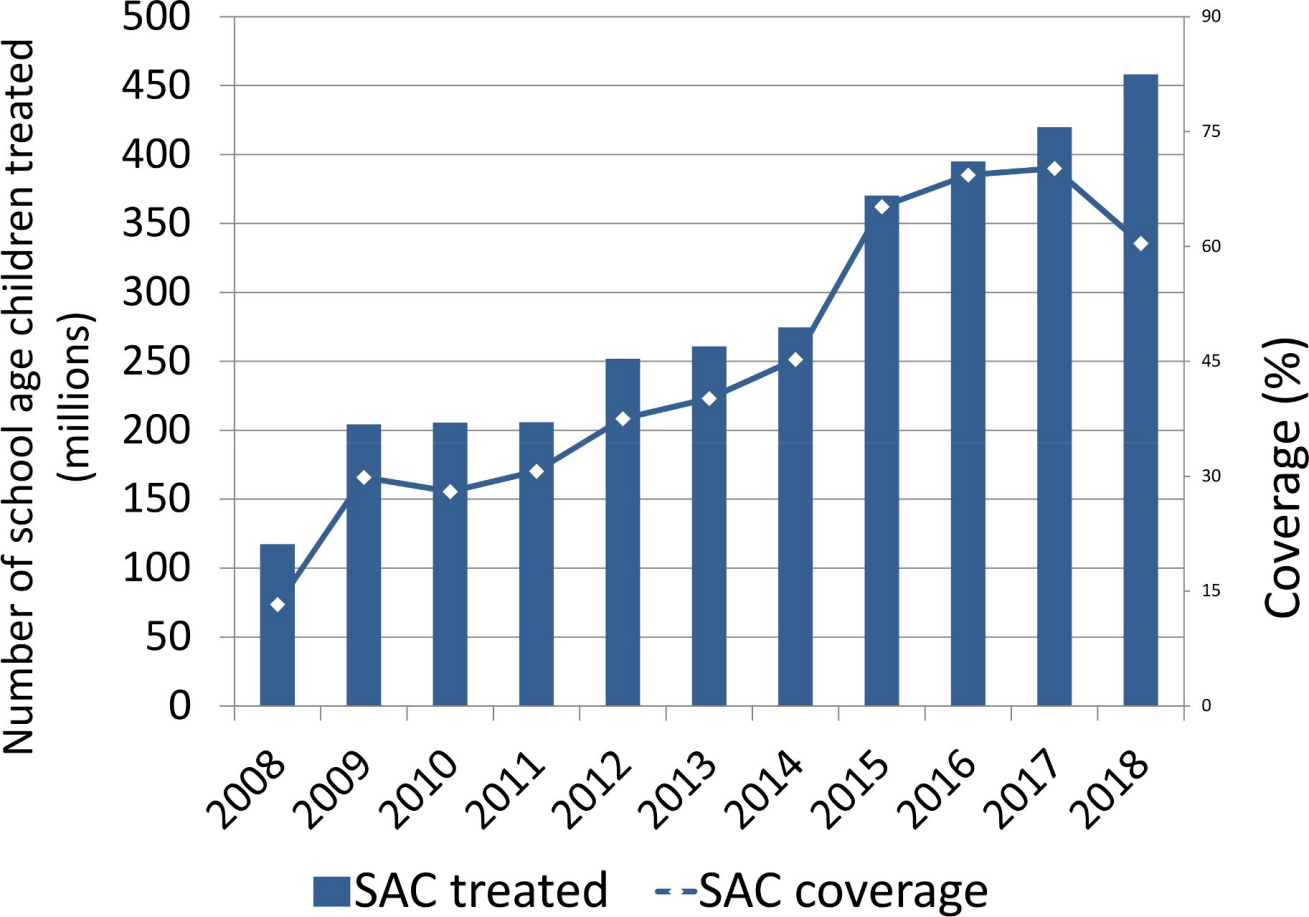
Progress in PC coverage of school-age children for STH between 2010 and 2018. Data are presented globally and stratified by WHO region (AFR = African Region; AMR = Region of the Americas; EMR = Eastern Mediterranean Region; EUR European Region; SEAR = South East Asian Region; WPR Western Pacific Region).

The reduction in global coverage observed in 2018, to 60% (Fig 3), occurred largely because of the decision by India, the largest country in terms of number of children in need of PC for STH, to further expand their program to areas previously considered not in need of PC. This expansion was substantial and resulted in a 23% increase in the denominator used to calculate global coverage, reducing the overall coverage rate. Hence, despite a global increase of 164 million additional school-age children being treated in 2018 (compared to 2017), the global coverage decreased from 70% to 60%. Globally, it is expected that PC coverage rates will further improve in future years as more endemic countries scale up their PC programs. Yet, the ongoing coronavirus disease 2019 (COVID-19) pandemic might jeopardize these developments.

Of the 96 countries where school-age children were considered in need of PC for STH infections in 2018, 28 countries reached effective coverage (≥75%) for more than 5 years; 42 countries reached the PC effective coverage, but for less than 5 years; 23 countries (including Armenia) established an STH control program but failed to reach the 75% PC coverage target; and 3 countries had not yet started or reported a PC program. Armenia, despite having implemented PC for less than 5 years, has been removed from the list of STH-endemic countries as of 2019, as recent surveys conducted by the MOH suggest that STH prevalence and intensity of infection are extremely low and is no longer of public health importance in the country.

Table 1 provides the status of the progress of PC implementation for school-age children in the endemic countries. Of the 28 countries that have implemented PC for STH with effective coverage in school-age children for more than 5 years, 17 have already conducted an impact assessment and two have stopped PC (i.e., Burkina Faso and Mali) although still maintaining surveillance to identify any possible rebound (Table 2).

**Table 1:** STH-endemic countries categorized by the progress of their PC implementation program as of 2018 for school-age children and total number in each category. Effective coverage is defined as a national coverage of over 75%. *Armenia will be removed from the list of STH-endemic countries as of 2019 following results of recent surveys documenting extremely low prevalence and infection intensity.

PC not started or not reported	PC implemented with no effective coverage	PC implemented with effective coverage less than 5 years	PC implemented with effective coverage over 5 years
Antigua and Barbuda	Angola	Benin	Afghanistan
Argentina	Armenia*	Brazil	Azerbaijan
Botswana	Bolivia	Cabo Verde	Bangladesh
	CAR	Congo	Bhutan
	Chad	Dominica	Burkina Faso
	Colombia	El Salvador	Burundi
	Comoros	Eq Guinea	Cambodia
	DRC	Fiji	Cameroon
	Djibouti	Gambia	Côte d'Ivoire
	Ethiopia	Guatemala	DPR Korea
	Gabon	Guinea	Dominican R
	Georgia	Guyana	Ghana
	Guinea-Bissau	Honduras	Haiti
	Indonesia	India	Kiribati
	Kenya	Iraq	Kyrgyzstan
	Namibia	Lesotho	Lao PDR
	Pakistan	Liberia	Malawi
	Papua New Guinea	Madagascar	Mali
	Popular Republic China	Marshall Islands	Mexico
	Sao Tome–Principe	Micronesia	Myanmar
	Solomon Islands	Mozambique	Nicaragua
	Sudan	Nauru	Rwanda
	South Sudan	Nepal	Sierra Leone
		Niger	Eswatini
		Nigeria	Tajikistan
		Panama	Togo
		Paraguay	Tuvalu
		Peru	Uganda
		Philippines	
		Senegal	
		Somalia	
		South Africa	
		Timor-Leste	
		Tonga	
		UR Tanzania	
		Uzbekistan	
		Vanuatu	
		Venezuela	
		Viet Nam	
		Yemen	
		Zambia	
		Zimbabwe	
**3**	**23**	**42**	**28**

**Table 2:** Status of countries that have implemented more than 5 years of PC for STH in school-age children, categorized by in terms of impact assessment and ongoing surveillance.

PC implemented with effective coverage over 5 years no impact assessment	PC implemented with effective coverage over 5 years and impact assessment conducted	PC suspended with surveillance activities on-going
Azerbaijan	Afghanistan	Burkina Faso
Côte d'Ivoire	Bangladesh	Mali
DPR Korea	Bhutan	
Dominican Republic	Burundi	
Kiribati	Cambodia	
Kyrgyzstan	Cameroon	
Malawi	Ghana	
Eswatini	Haiti	
Tajikistan	Lao PDR	
Tuvalu	Mexico	
Uganda	Myanmar	
	Nicaragua	
	Rwanda	
	Sierra Leone	
	Togo	
**11**	**15**	**2**

#### Women of reproductive age

Women of reproductive age is the third population group at highest risk of STH-attributable morbidity. Thus far, there is no formal reporting process to estimate coverage of anthelmintics in this at-risk group. However, a recent analysis of data from Demographic and Health Surveys (DHS) of over 700,000 women in 49 countries estimates that approximately 25% have received PC for STH during their last pregnancy [14]. Additionally, approximately 140 million women of reproductive age are treated annually with albendazole in the context of the global program for the elimination of lymphatic filariasis (GPELF) corresponding to an approximate coverage of 20% [10]. WHO recommends that the STH epidemiologic situation be evaluated when a transmission assessment survey (TAS) for lymphatic filariasis is being conducted in order to take advantage of the data collection activities in the field and then, to make a decision on which following steps would be required for STH control [15].

## Part 2: Impact of PC and other control activities on STH prevalence and infection intensity

The impact of PC on STH can be assessed in a variety of ways. Endemic countries are invited to evaluate their progress periodically with a survey (at least after 5 years of PC intervention) and to report to WHO. The results of these surveys provide an evaluation of the impact of the intervention in terms of a reduction in STH-attributable morbidity, where morbidity is inferred from infections of moderate and heavy intensity (intensity being measured in terms of the number of parasite eggs per gram of feces). Indeed, the prevalence of moderate and heavy intensity STH infections is the recommended indicator to assess whether the ultimate goal of eliminating STH as public health problem has been achieved (i.e., the prevalence of infection of moderate and heavy intensity is under 1%) [16]. A recent review of data from 15 countries that had implemented PC for school-age children for more than 5 years showed that infections of moderate and heavy intensity had been almost completely eliminated [17] and that prevalence was reduced by two-thirds [18].

Two recent Cochrane reviews of markedly heterogeneous sets of randomized trials of deworming in children concluded that there was limited improvement in terms of nutritional indicators, hemoglobin level, and children’s performance in school [19,20]. However, the methodology used in these reviews has been criticized (i.e., among other limitations was the failure to take into account the fact that no health benefits would be expected to be accrued to uninfected individuals in a deworming program. Hence, if the average health benefits are measured on the entire population treated, a dilution effect would result in observing reduced benefits accrued in infected individuals [21–24]. An independent review commissioned by WHO [25] recommended deworming of high-risk groups as a cost-effective community-based intervention noting the following key points: (i) STH infection can cause important morbidity; (ii) the anthelmintic drugs used in PC are effective in reducing STH morbidity; (iii) adverse events caused by the administration of the anthelmintic drugs are few, mild, transient, and reversible; and (iv) the most cost-effective way to reach infected individuals is via large scale distribution (after a survey establishing the STH prevalence level).

The conclusions of the WHO Guideline Development Group are confirmed by more recent studies: Montresor *et al*. [26] estimated that over 40% of the 1 262 000 disability-adjusted life years (DALYs) estimated to be lost annually due to STH in children [27] were averted in 2015 by PC programs. This study also estimated that, with the anticipated continued implementation of the program, STH-attributable morbidity in children would be reduced to nearly zero by 2025 [26].

In conclusion, the investment in the PC program globally implemented in the last decade has returned a substantial reduction in STH infection and associated morbidity. Caution must, however, be exercised in abruptly terminating PC interventions, as this may result in a rebound of prevalence and, consequently, in the loss of gains accrued, especially in populations without access to adequate sanitation.

In the absence of any substantial improvement in sanitation, WHO suggests a progressive reduction in the frequency of PC, coupled with a regular collection of epidemiologic data, to identify any possible prevalence rebound. A decision tree has been developed by WHO to assist program managers in making decisions on PC frequency [3]. The global effort to reduce poverty and improve access to sanitation (UN Sustainable Development Goals 1 (no poverty)and 6 (clean water and sanitation) [28] is expected to have an important impact on the transmission of STH, further reducing STH prevalence and attributable morbidity. However, at this time, it is difficult to predict the magnitude of this effect or its impact on STH infection outcomes.

## Part 3: The six targets recommended by WHO for STH prevention and control up to 2030

An international group of experts from STH-endemic countries, together with partners from institutions supporting STH prevention and control activities, met in Basel, Switzerland in October 2018 to identify STH control targets for 2030 [29]. Six targets were identified: (i) achieve and maintain elimination of STH morbidity in preschool- and school-age children; (ii) reduce the number of tablets needed in PC for STH; (iii) increase domestic financial support in PC for STH; (iv) establish efficient STH control programs in adolescent, pregnant and lactating women of reproductive age; (v) establish an efficient strongyloidiasis control program in school-age children; and (vi) ensure universal access to at least basic sanitation and hygiene in STH-endemic areas. The intentions behind these target activities and the indicators that are recommended to evaluate their progress and completion are briefly discussed below.

### Achieve and maintain elimination of STH-attributable morbidity in preschool- and school-age children

This target builds on the reduction in STH morbidity observed between 2010 and 2018 when a large majority of endemic countries established PC programs for STH prevention and control. The countries that started PC programmes early have already reached an important level of success in eliminating morbidity and reducing overall STH prevalence [17]. If PC implementation continues to scale up with the present trend, it is expected that most STH-endemic countries will eliminate STH-attributable morbidity in preschool- and school-age children by 2025 [26]. Benefit is also expected to be observed with the mebendazole donation from Johnson & Johnson, which, from 2020 onwards, covers not only school-age children, as initially set, but also preschool-age children [12].

### Reduce the number of tablets needed in PC for STH

It is expected that impact evaluation surveys will show a substantial reduction in global STH prevalence and intensity, and that, with time, this will translate into a reduction in the frequency of the PC intervention and, as a consequence, a reduction in the number of tablets needed to maintain the PC programs. A reduction in the number of tablets needed is not expected to occur uniformly within a country. It may be that areas where transmission is particularly high would continue with the present pace of PC administration to prevent a rebound in prevalence and intensity of STH infections.

The global reduction in tablet need is estimated to be on the order of 49% by 2030 [30]. One consequence will be a surplus of donated tablets that could eventually be used to expand PC to other at-risk groups.

### Increase domestic financial support to PC for STH

The reduction in the number of PC rounds needed will also further reduce the cost of PC programs and offer the possibility for some STH-endemic countries to increase domestic financial support for their PC programs, thus facilitating also the achievement of Universal Health Coverage (UHC). Marocco *et al*. [30] estimated that 28 STH-endemic countries in the World Bank's upper-middle income country group with annual per capita health spending over US$ 461 could afford the cost of PC to respond to possible reduced anthelmintic efficacy (estimated to be on the order of US$ 0.50 per person per year in need of treatment). However, countries in the low-middle income group are not expected to become independent in treatment procurement by 2030 and for them the donation of benzimidazoles is expected to be maintained.

### Establish an efficient STH control program in adolescent, pregnant, and lactating women

Women of reproductive age are the third group at high risk of STH-attributable morbidity. However, to date, this group has been the most neglected in terms of targeted PC to control STH. Although no dedicated reporting system is yet in place to collect data on PC coverage in women of reproductive age, indirectly, using reports from DHS, it has been estimated that approximately 20% of pregnant women receive PC for STH during pregnancy and coverage appears to be higher in African countries [14]. This shows the feasibility of undertaking a simple intervention (i.e., provision of anthelmintics for STH in first level health care services during or shortly following a pregnancy) even in resource-limited settings. WHO therefore recommends that all STH-endemic countries provide anthelmintics to at-risk pregnant women by 2030.

With this target in mind, in 2020, the WHO Department of Control of Neglected Tropical Diseases is developing a policy paper outlining the evidence in support of PC for pregnant women. The essential principle is that, if a woman over her entire lifespan receives deworming regularly (i.e., during preschool (1–4 years), in school (5–14 years), during adolescence (15–19 years), and at antenatal and postpartum health services, then cumulatively, these interventions would be sufficient to keep STH morbidity low in this risk group.

### Establish an efficient strongyloidiasis control program in school-age children

*S*. *stercoralis* infection may cause dermatological and gastro-intestinal morbidity, chronic malnutrition in children, and even mortality in immunocompromised patients [31]. The drug of choice for treating this infection is ivermectin given as a single dose [32]. In areas where ivermectin has been distributed as PC for the elimination of lymphatic filariasis or onchocerciasis, strongyloidiasis prevalence and related morbidity have dramatically decreased [33,34]. The practical implementation of PC for this infection has been hampered by difficulties in accessing ivermectin of good quality at an affordable price and by the lack of readily available diagnostic approaches with high sensitivity and specificity (in comparison to other STHs) [35]. At present, ivermectin is donated only for the elimination of lymphatic filariasis and onchocerciasis, but recently, WHO invited generic producers of ivermectin to submit their formulation for pre-qualification such that an affordable generic form of ivermectin may be expected to be available soon. Moreover, the global prevalence of strongyloidiasis has long been underestimated due to the poor sensitivity of the diagnostic methods that are usually used for assessing the prevalence of STH [36]. Recent estimates based on mathematical models, accounting for the (in)accuracy of the diagnostics used for prevalence surveys suggest a much higher prevalence than previously estimated [37].

As the knowledge-base on the global epidemiology of *S*. *stercoralis* improves, it may be that PC for this parasite infection is found to be warranted in more countries. In this case, the existing infrastructure could also provide ivermectin to school-age children [37]. This school-based intervention is not aimed at completely solving the problem of strongyloidiasis (which includes morbidity also in adults), but at providing a minimal intervention utilizing existing infrastructure and low-cost medicines. In other words, to do the maximum that can be done with the available resources [38]. A more comprehensive intervention including adults is certainly appropriate, but costs must be taken into account. The cost of reaching adults with PC has been estimated to be more than 10 times that of reaching school-age children [39,40]. Therefore, a comprehensive approach for strongyloidiasis control would need substantial funding that is currently not available.

### Ensure universal access to at least basic sanitation and hygiene by 2030 in STH-endemic areas

Adequate sanitation is essential to maintain the benefits of PC and to provide the long-term foundation for the elimination of STH morbidity. PC alone is not sufficient to eliminate STH in endemic areas, for the following two reasons. First, STHs have a remarkable capacity to contaminate the environment with high numbers of eggs and larvae (e.g., in a soil survey conducted in The Philippines in an area with a STH prevalence of 44%, STH eggs were found in 85% of the soil samples collected in the house, in the back yard, or on the footpaths around the village [41]). Second, STH eggs can survive in the environment for prolonged periods of time; several weeks in the case of hookworms [42], a few years in the case of *T*. *trichiura* [43] and more than 10 years in the case of *A*. *lumbricoides* [44]. For these two reasons, in intensively contaminated environments, opportunities for STH transmission can persist even in the absence of current infection in the host population and it will be impossible to eliminate the risk of reinfection with an intervention that targets only the human reservoir [4].

To corroborate this situation, it has been observed that, in all previously endemic countries where STH transmission was considerably reduced and morbidity permanently eliminated, an efficient sanitation infrastructure was put in place, preventing the possibility of environmental contamination with human excreta and greatly reducing human contact with fecal matter [45].

The Department of Control of Neglected Tropical Diseases at WHO is promoting coordination and joint planning with programs aimed at improving standards of sanitation (e.g., the sharing of epidemiologic data that can help identify areas in need of sanitation; mapping prevalence data alongside sanitation coverage; providing input into water, sanitation, and hygiene (WASH) program design; and promoting interventions for behavioral change within school health curricula). Documents identifying examples for possible areas of collaboration have been developed jointly by the Department of Control of Neglected Tropical Diseases and the Department of Public Health, Environmental and Social Determinants of Health [46].

## Discussion

To achieve the 2030 targets for STH control, the following tools and activities are needed: (a) Manuals and training to support countries in evaluating the impact of the control measures implemented. A rapid method to assess impact is needed; A geo-statistic approach is currently under development to reduce the required sample size, and therefore the cost, of carrying out an impact survey. If proven successful, a manual and training activities on this approach will be put in place. (b) Updated manual for guiding countries in the evaluation of drug efficacy. The standard protocol to assess anthelmintic drug efficacy was developed by WHO in 2012 [47]. An update, especially regarding the possible steps to take in case of reduced efficacy, is needed. (c) Additional anthelmintic drugs, or combination of existing anthelminthics. Additional drugs may not necessarily need to be new. There are a number of anthelmintic drugs which have been developed in the past that are not in use because of the superiority of the benzimidazoles. These drugs should be re-evaluated and their use in combination employed to increase the spectrum of activity. For example, should there emerge resistance in *T*. *trichiura* infections, it is already possible to add ivermectin to either benzimidazole. In addition, this combination would also be efficacious in treating *S*. *stercoralis* infection. (d) A strategy to control STH in women of reproductive age. A general approach to the treatment of this group at risk has been problematic. In reality, this group is composed of four distinct subgroups: (i) adolescent girls; (ii) pregnant women; (iii) lactating women; and (iv) non-pregnant non-lactating women [7]. Each of these sub-groups has different characteristics, which necessitate different approaches to treatment. A strategy that focuses on those sub-groups that are more easily reachable through the health care system (i.e., pregnant and lactating women) would probably be more successful in the short term. WHO is modeling the possible impact of this approach. Ultimately, efforts should also be made to ensure access to anthelmintics for all four sub-groups of this at-risk group. (e) Increased access to ivermectin. Good quality ivermectin at an affordable price should be available to control strongyloidiasis. (f) Pilot programs for control of strongyloidiasis. At the moment the possibility to control strongyloidiasis has been evaluated only theoretically or indirectly (i.e., impact of PC with ivermectin distributed for other purposes) [4,33,34]. The proposed approach for controlling strongyloidiasis should be field tested and the results documented. (g) Improved diagnostics.

Innovative serological diagnostic approaches (e.g., rapid, field-applicable tests) for *A*. *lumbricoides*, *T*. *trichiura*, and hookworm infections have proven difficult to develop because of the number of different species involved (that increases the possibility of cross-reactivity) and the need for managers of control programs to measure, not only the presence of STH infections, but also their intensity. The diagnostic approach presently used for STH is based on microscopic evaluation of fecal specimens, a labour-intensive method, which requires specially trained laboratory personnel. A variety of computerized and automated approaches to facilitate and speed up the accurate reading of microscopic slides are presently in development and are expected to reduce the time and the number of personnel needed for the diagnosis.

WHO is fully committed to engage with all concerned partners, and specifically with partners in the WASH sector, in order that the necessary tools, guidance, and support is provided to all STH-endemic countries so that the ultimate goal of eliminating morbidity due to STH and strongyloidiasis is achieved by 2030.
